# Relevance of suspicious lymph nodes in preoperative imaging for resectability, recurrence and survival of intrahepatic cholangiocarcinoma

**DOI:** 10.1186/s12893-020-00730-x

**Published:** 2020-04-15

**Authors:** Fabian Bartsch, Felix Hahn, Lukas Müller, Janine Baumgart, Maria Hoppe-Lotichius, Roman Kloeckner, Hauke Lang

**Affiliations:** 1 Department of General, Visceral and Transplant Surgery, University Medical Center of the Johannes Gutenberg-University Mainz, Langenbeckst. 1, 55131 Mainz, Germany; 2 Department of Diagnostic and Interventional Radiology, University Medical Center of the Johannes Gutenberg-University Mainz, Mainz, Germany

**Keywords:** Intrahepatic cholangiocarcinoma, Cholangiocarcinoma, Preoperative imaging, Lymph node, recurrence, survival

## Abstract

**Background:**

Intrahepatic cholangiocarcinoma (ICC) is often diagnosed at an advanced stage resulting in a low resectability rate. Even after potentially curative resection the risk for tumor recurrence is high. Although the extent and value of lymphadenectomy is part of ongoing discussion, the role of preoperative imaging for assessment of suspicious lymph nodes (suspLN) has only been studied modestly. Aim of this study is to demonstrate the influence of suspicious lymph nodes in preoperative imaging on resectability, recurrence, and long-term outcome.

**Methods:**

All patients who underwent exploration for ICC between January 2008 and June 2018 were included. Preoperative imaging (CT or MRI) was analysed with focus on suspLN at the hepatoduodenal ligament, lesser curvature, interaortocaval, and superior to the diaphragm; suspLN were classified according to the universally accepted RECIST 1.1 criteria; histopathology served as gold standard.

**Results:**

Out of 187 patients resection was performed in 137 (73.3%), in 50 patients the procedure was terminated after exploration. Overall, suspLN were found preoperatively in 73/187 patients (39%). Comparing patients who underwent resection and exploration only, suspLN were significantly more common in the exploration group (*p* = 0.011). Regarding lymph node stations, significant differences could be shown regarding resectability: All tumors with suspLN superior to the diaphragm were irresectable. Preoperative imaging assessment showed a strong correlation with final histopathology, especially of suspLN of the hepatoduodenal ligament and the lesser curvature. Sensitivity of suspLN was 71.1%, specificity 90.8%. Appearance of tumor recurrence was not affected by suspLN (*p* = 0.289). Using a short-axis cut-off of <> 1 cm, suspLN had significant influence on recurrence-free survival (RFS, *p* = 0.009) with consecutive 1-, 3-, and 5-year RFS of 41, 21, and 15% versus 29, 0, and 0%, respectively. Similarly, 1-, 3- and 5-year overall survival (OS) was 75, 30, and 18% versus 59, 18, and 6%, respectively (*p* = 0.040).

**Conclusion:**

Suspicious lymph nodes in preoperative imaging are predictor for unresectability and worse survival. Explorative laparoscopy should be considered, if distant suspicious lymph nodes are detected in preoperative imaging. Nevertheless, given a sensitivity of only 71.1%, detection of suspicious lymph nodes in the preoperative imaging alone is not sufficient to allow for a clear-cut decision against a surgical approach.

## Background

Harvesting of lymph nodes is established in many different malignancies and the 8th edition of the AJCC/UICC/TNM classification recommends a specific number of received lymph nodes per entity [1]. For intrahepatic cholangiocarcinoma (ICC) the importance and extension of lymphadenectomy is still in debate with a recommended number of six lymph nodes to be harvested currently. In different studies tumor positive lymph nodes had significant influence on long-term outcome [2–8]. In the recent literature factors regarding lymphadenectomy such as the total number of harvested lymph nodes, the number of malignant lymph nodes or positive LN to negative LN ratios are discussed extensively [9–11]. ICC is commonly diagnosed in an advanced tumor stage due to missing or late occurrence of symptoms. Complete resection offers the only chance of cure. The number of negative laparotomies and unresectability is high with resection rates of only 50 to 75% [7, 8, 12, 13]. Long-term outcome is determined by tumor recurrence, which is common in 40–76% of patients [7, 14, 15]. Precise prediction of resectability and/or prognosis is often difficult based on preoperative imaging. If extrahepatic spread is diagnosed, palliative treatment is initiated in most cases. If the ICC appears technically resectable, most often suspicious or borderline suspicious lymph nodes are discussed as lymph node metastasis have been shown to be highly predictive [16]. However, only a few publications regarding preoperative imaging and the aspect of lymph nodes are available for ICC. One study showed a positive predictive value for metastatic disease of 67% in round lymph nodes exceeding 18 mm which are rare [17]. Another study used positron emission tomography-computed tomography (PET-CT) and concluded that preoperative evaluation remains difficult [18] while a recent meta-analysis demonstrated PET-CT imaging to be accurate in the evaluation of primary tumors, lymph node and distant metastasis [19]. The most recent article found lymph node enlargement with a small axis diameter exceeding 1 cm in preoperative imaging as the strongest predictor for histopathological lymph node metastases [20].

Hence, the aim of this study is to analyse the preoperative imaging in four lymph node stations and the influence of suspLN on resectability, recurrence and long-term outcome.

## Methods

All patients who underwent exploration because of ICC between January 2008 and June 2018 were prospectively collected in an institutional database and the data was transferred to SPSS 23 (SPSS Inc., released 2014, IBM SPSS Statistics for Windows, Version 23.0, IBM Armonk, NY, USA: IBM Corp). Patients with secondary liver malignancies as well as hepatocellular carcinoma, perihilar cholangiocarcinoma and gallbladder carcinoma were excluded. Further analyses were performed retrospectively and only patients with complete datasets were included.

All patients signed informed consent that data and follow-up will be collected anonymously and potentially used for scientific analysis. Regarding to the regulations of the federal state law (state hospital law §36 & §37) and the independent ethics committee of Rhineland-Palatinate, no ethical approval was necessary for this study. The work has been carried out in accordance with the Declaration of Helsinki.

### Preoperative evaluation and imaging

For preoperative staging, we preferred contrast enhanced multiphasic computed tomography (CT). Many patients presented with the initial diagnosis already made at referring centers or in an ambulant setting. We accepted externally produced CT or magnetic resonance imaging (MRI) if it was of sufficient quality.

Lymph nodes were classified preoperatively as suspicious according to the original RECIST 1.1 criteria, the RECIST 1.1 amendment specifically designed for lymph node assessment and further supporting literature [21, 22]. Criteria applied to determine malignancy were first and foremost the size with a short-axis diameter >  1.5 cm but also further ancillary imaging features such as spherical shape, no fatty hilus, strong contrast enhancement, MR-diffusion restriction, central necrosis, threshold growth and distribution pattern [23]. Examined lymph node stations were the hepatoduodenal ligament, retropancreatic, the lesser curvature, interaortocaval, and superior to the diaphragm. The axial diameter of the largest suspicious lymph node of each of the four stations was gathered.

### Surgery, postoperative outcome and follow-up

All explorations were performed by a group of experienced surgeons with special expertise in liver surgery. We have an aggressive attitude regarding resection at our center and perform visceral and vascular resections and reconstructions if reasonable to achieve complete tumor clearance. Suspicious lymph nodes were no reason to resign from resection if they were found in lymph node stations close to the liver. If suspicious lymph nodes were found distantly for example interaortocaval, several considerations were made regarding perioperative risk and constitution of the patient. In selected cases frozen section was performed, and if distant lymph node metastasis were detected we resigned from resection. Intra- and postoperative parameters were collected like type of resection, histological results, morbidity, mortality, and tumor recurrence. Lymphadenectomy was performed routinely in case of ICC at least in the hepatoduodenal ligament. After the new 8th edition of the UICC classification was published in 2017 lymphadenectomy was performed according to the included recommendations: for ICC of the right hepatic lobe lymphadenectomy contained the hepatoduodenal ligament, peripancreatic and periduodenal lymph nodes; for ICC of the left hepatic lobe lymphadenectomy contained the hepatoduodenal ligament and gastrohepatic lymph nodes [1]. Nevertheless, a small subset of patients had a Nx situation (*n* = 16). Although a lymphadenectomy was performed, no lymph nodes could be detected by the pathologist (*n* = 5). If hepatocellular carcinoma was suspected based on imaging, no lymphadenectomy was performed and the diagnosis of ICC was made through the final histology (*n* = 11).

Follow-up was conducted every 3 months after initial surgery for at least two years; later on the interval was increased to 6 months, if reasonable. CT or MR imaging was carried out at least every 6 months in turns with ultrasound examinations. If patients were not able to undergo follow-up at our center, for example because of logistic reasons, we stayed in contact with the treating physician to obtain all information needed. All patients signed informed consent for this kind of follow-up.

### Statistical analyses

Categorical data was analysed using the Chi^2^ test in cross tabulation. Survival analyses were conducted with Kaplan-Meier curves and log rank test. A *p*-value of < 0.05 was considered significant. All analyses were intention-to-treat. Recurrence-free survival was defined according to Punt and colleagues [24].

## Results

We report on 187 patients who underwent 137 resections (73.3%) and 50 explorations. Patients’ demographics, surgical characteristics and reasons for unresectability are provided in Table 1. Anatomical major resections were performed in 68.6%. Morbidity after resection appeared in 38.7%, the 90-day mortality was 8%.

**Table 1:** Patients characteristics

All patients	***n*** = 187
**Age** [Median, (IQR)] Range	63.8 (55.8–73.7) 32.3–84.4
**Gender** [Female / Male]	93 / 94
**ASA classification**	
ASA I	2
ASA II	79
ASA III	102
ASA IV	4
**Resections**	***n*** **= 137**
Right trisectionectomy	25
Left trisectionectomy	18
Right hepatectomy	22
Left hepatectomy	16
Mesohepatectomy ^a^	7
ALPPS	6
Monosegmentectomy	9
Bisgementectomy	23
Resection of three liver sg.	9
Atypic / wedge resection	2
**Vascular resections / reconstruction**	
Portal vein / major liver vein / inferior vena cava	17 / 19 / 13
**Visceral resections / reconstruction**	
Adren / Diaphragm / Duodenum / Stomach / Colon	4 / 9 / 1 / 1 / 1
**Histological outcome (8th edition of UICC classif.)**	
T1a	22
T1b	30
T2	54
T3	12
T4	19
N0	82
N1	39
NX	16
M0	129
M1 ^b^	8
R0	123
R1	14
G1 – well	3
G2 – moderate	85
G3 – poor	38
G4 – undifferentiated	1
No grading – neoadjuvant therapy	10
UICC Ia	14
UICC Ib	19
UICC II	34
UICC IIIa	6
UICC IIIb	41
UICC IV	7
Cirrhosis	9^c^
**Unresectable**	***n*** **= 50**
Peritoneal carcinomatosis	19
Multifocal spread	11
Cirrhosis	3
small/impaired FLR^d^	7
Complex infiltration / advanced	10

^a^ Central resections ≥3 segments; ^b^ in these patients incidentally localized peritoneal spread was diagnosed in final histology; ^c^ all patients with cirrhosis underwent minor resections (bisegment *n* = 7; monosegment n = 1, atypic resection n = 1); ^d^*FLR* future liver remnant; 16 NX patients have no UICC stadium

Suspicious lymph nodes within the preoperative imaging were found in 74 cases (39.6%) of the entire cohort. This divides further in 47 out of 137 patients (34.3%) in the resection group and 27 out of 50 patients (54%) in the exploration group which showed a statistically significant difference between these two groups (*p* = 0.015).

### Dissemination of suspicious lymph nodes and influence on resectability

The dissemination of suspLN is shown in Table 2. Overall or in any analysed lymph node station the distribution of suspicious lymph nodes showed to be significantly influence resectability.

**Table 2:** Dissemination of suspicious lymph nodes

	All ***n*** = 187	Resection group ***n*** = 137	Exploration group ***n*** = 50	***p***-value
**Suspicious lymph nodes**^**a**^				
Yes	74 (39.6%)	47 (34.3%)	27 (54%)	**0.015**
No	113 (60.4%	90 (65.7%)	23 (46%)	
**SuspLN hepatoduodenal lig.**				
Yes	68 (36.4%)	43 (31.4%)	25 (50%)	**0.019**
No	119 (63.6%)	94 (68.6%)	25 (50%)	
**SuspLN retropancreatic**				
Yes	25 (13.4%)	12 (8.8%)	13 (26%)	**0.002**
No	162 (86.6%)	125 (91.2%)	37 (74%)	
**SuspLN lower curvature**				
Yes	33 (17.6%)	17 (12.4%)	16 (32%)	**0.002**
No	154 (82.4%)	120 (87.6%)	34 (68%)	
**SuspLN interaortocaval**				
Yes	22 (11.8%)	10 (7.3%)	12 (24%)	**0.002**
No	165 (88.2%)	127 (92.7%)	38 (76%)	
**SuspLN supradiaphragmal**				
Yes	3 (1.6%)	0 (−)	3 (6%)	**0.004**
No	184 (98.4%)	137 (100%)	47 (94%)	

^a^ overall in any lymph node station; *SuspLN* Suspicious lymph nodes

Comparing the influence on resectability for all five lymph node stations distinguished by size of the suspLN statistical significance is reached as well (Table 3). To demonstrate the amount of suspLN of the exploration group a ratio was calculated (suspLN exploration group/suspLN of lymph node station). The ratio raises analogously from the hepatoduodenal ligament with 36.8% (25/68), the lower curvature with 48.5% (16/33), retropancreatic with 52% (13/25), interaortocaval with 54.5% (12/22) to superior of the diaphragm with 100% (3/3).

**Table 3:** Distribution of suspicious lymph nodes distinguished by size

	All	Resection group	Exploration group	***p***-value
**All LN stations together**	*n* = 935^a^	*n* = 685	*n* = 250	
No suspicious LN	784 (83.9%)	603 (88%)	181 (72.4%)	
< 1 cm	63 (6.7%)	39 (5.7%)	24 (9.6%)	
1–1.5 cm	71 (7.6%)	37 (5.4%)	34 (13.6%)	
> 1.5 cm	17 (1.8%)	6 (0.9%)	11 (4.4%)	
**Hepatoduodenal lig.**	*n* = 187	*n* = 137	*n* = 50	
No suspicious LN	119 (63.6%)	94 (68.6%)	25 (50%)	**p = 0.001**
< 1 cm	22 (11.8%)	18 (13.1%)	4 (8%)	
1–1.5 cm	33 (17.6%)	21 (15.3%)	12 (24%)	
> 1.5 cm	13 (7%)	4 (3%)	9 (18%)	
**Retropancreatic**	*n* = 187	*n* = 137	*n* = 50	
No suspicious LN	162 (86.6%)	125 (91.3%)	37 (74%)	**p = 0.009**
< 1 cm	9 (4.8%)	4 (2.9%)	5 (10%)	
1–1.5 cm	16 (8.6%)	8 (5.8%)	8 (16%)	
> 1.5 cm	–	–	–	
**Lower curvature**	*n* = 187	*n* = 137	*n* = 50	
No suspicious LN	154 (82.3%)	120 (87.6%)	34 (68%)	**p = 0.009**
< 1 cm	16 (8.6%)	9 (6.6%)	7 (14%)	
1–1.5 cm	16 (8.6%)	7 (5.1%)	9 (18%)	
> 1.5 cm	1 (0.5%)	1 (0.7%)	–	
**Interaortocaval**	*n* = 187	*n* = 137	*n* = 50	
No suspicious LN	165 (88.2%)	127 (92.7%)	38 (76%)	***p*** **= 0.004**
< 1 cm	14 (7.5%)	8 (5.9%)	6 (12%)	
1–1.5 cm	6 (3.2%)	1 (0.7%)	5 (10%)	
> 1.5 cm	2 (1.1%)	1 (0.7%)	1 (2%)	
**Supradiaphragmal**	*n* = 187	*n* = 137	*n* = 50	
No suspicious LN	184 (98.4%)	137 (100%)	47 (94%)	**p = 0.015**
< 1 cm	2 (1.1%)	–	2 (4%)	
1–1.5 cm	–	–	–	
> 1.5 cm	1 (0.5%)	–	1 (2%)	

^a^ Five lymph node stations per 187 patients

### Suspicious lymph nodes in relation to final histopathological results

A median of five lymph nodes were harvested (IQR 1–7, range 0–31) within the resection group and N0 status was achieved in 82 patients. In the final histopathological report, 39 patients were classified with N1 disease and 16 patients were classified Nx without any lymph nodes harvested. In correlation of suspLN through imaging and histopathologic result sensitivity was 71.1% and specificity 90.8%. The presence of suspLN in preoperative imaging had a significant influence on the nodal status with a higher likelihood to have N+ status, either for differentiation in suspLN present (or not) or different diameter of suspicious nodes (*p* < 0.001, each; see Table 4). Presence of suspLN had significant influence on nodal status for lymph nodes of the hepatoduodenal ligament (*p* < 0.001), retropancreatic (*p* = 0.011) and the lower curvature (*p* = 0.001), while interaortocaval lymph nodes had not (*p* = 0.540).

**Table 4:** Relation of preoperative imaging and histopathological nodal status

**All lymph node stations**					
**Suspicious nodes**	**n**	**N1**	**N0**	**N1/N0**^**a**^	
No	76 (62.8%)	7 (17.9%)	69 (84.1%)	0.10	**p < 0.001**
< 1 cm	17 (14%)	9 (23.1%)	8 (9.8%)	1.13	
1–1.5 cm	22 (18.2%)	18 (46.2%)	4 (4.9%)	4.5	
> 1.5 cm	6 (5%)	5 (12.8%)	1 (1.2%)	5	
No suspicious nodes	76 (62.8%)	7 (17.9%)	69 (84.1%)	0.10	**p < 0.001**
Suspicious nodes (any size)	45 (37.2%)	32 (82.1%)	13 (15.9%)	2.5	
**Hepatoduodenal ligament**					
**Suspicious nodes**	**n**	**N1**	**N0**	**N1/N0**^**a**^	
No	79 (65.3%)	9 (23.1%)	70 (85.3%)	0.13	**p < 0.001**
< 1 cm	17 (14%)	9 (23.1%)	8 (9.8%)	1.13	
1–1.5 cm	21 (17.4%)	17 (43.5%)	4 (4.9%)	4.25	
> 1.5 cm	4 (3.3%)	4 (10.3%)	–	4	
**Retropancreatic**					
**Suspicious nodes**	**n**	**N1**	**N0**	**N1/N0**^**a**^	
No	110 (90.9%)	31 (79.5%)	79 (86.3%)	0.39	**p = 0.011**
< 1 cm	4 (3.3%)	3 (7.7%)	1 (1.2%)	3	
1–1.5 cm	7 (5.8%)	5 (12.8%)	2 (2.4%)	2.5	
> 1.5 cm	–	–	–	X	
**Lower curvature**					
**Suspicious nodes**	**n**	**N1**	**N0**	**N1/N0**^**a**^	
No	105 (86.8%)	26 (66.6%)	79 (86.3%)	0.33	**p = 0.001**
< 1 cm	8 (6.6%)	6 (15.4%)	2 (2.4%)	3	
1–1.5 cm	7 (5.8%)	6 (15.4%)	1 (1.2%)	6	
> 1.5 cm	1 (0.8%)	1 (2.6%)	–	X	
**Interaortocaval**					
**Suspicious nodes**	**n**	**N1**	**N0**	**N1/N0**^**a**^	
No	112 (92.6%)	34 (87.1%)	78 (95.1%)	0.44	p = 0.540
< 1 cm	7 (5.8%)	4 (10.3%)	3 (3.7%)	1.33	
1–1.5 cm	1 (0.8%)	1 (2.6%)	–	X	
> 1.5 cm	1 (0.8%)	–	1 (1.2%)	X	

NX patients were excluded (n = 16); ^**a**^ ratio between positive and negative nodal status (X if ratio is mathematically not possible)

A correlation of clinicopathological parameters with suspLN and N status showed a significant influence of T and N status as well as UICC stage and suspLN (p < 0.001 each) as demonstrated in Table 5. Tumor size, multifocal disease and number of nodules had no significant effect.

**Table 5:** Clinicopathological parameters in correlation with suspLN and N status

		suspLN			N status^**a**^		
	n = 137	yes	no	***p***-value	N0	N+	***p***-value
**Tumor size**^**b**^							
≥ 5 cm	99 (72.3%)	38 (27.7%)	61 (44.5%)	0.105	63 (52.1%)	26 (21.5%)	0.236
< 5 cm	38 (27.7%)	9 (6.6%)	29 (21.2%)		19 (15.7%)	13 (10.7%)	
**Multifocal disease**							
No – solitary	96 (70.1%)	30 (21.9%)	66 (48.2%)	0.249	56 (46.3)	26 (21.5%)	0.858
Yes	41 (29.9%)	17 (12.4%)	24 (17.5%)		26 (21.5%)	13 (10.7%)	
**Number of nodules**							
≤ 2	105 (76.6%)	32 (23.4%)	73 (53.3%)	0.087	64 (52.9%)	27 (22.3%)	0.294
≥ 3	32 (23.4%)	15 (10.9%)	17 (12.4%)		18 (14.9%)	12 (9.9%)	
**T status**							
T1 + 2	103 (75.2%)	32 (23.4%)	71 (51.8%)	0.165	69 (57%)	20 (16.5%)	**< 0.001**
T3 + 4	34 (24.8%)	15 (10.9%)	19 (13.9%)		13 (10.8%)	19 (15.7%)	
**UICC stage**^**a**^							
UICC I + II	67 (55.4%)	10 (8.3%)	57 (47.1%)	**< 0.001**	67 (55.4%)	–	**< 0.001**
UICC III + IV	54 (44.6%)	35 (28.9%)	19 (15.7%)		15 (12.4%)	39 (32.2%)	

^**a**^ NX patients were excluded (*n* = 16) or UICC was not applicable; ^b^ diameter of largest nodule

### Influence of suspicious lymph nodes on tumor recurrence and survival

Median overall survival (OS) of the resection group was 21.9 months with a consecutive 1-, 3- and 5-year OS of 72, 28, and 16%, respectively.

Recurrence of any kind occurred in 90 patients (65.7%) and suspLN in preoperative imaging had no influence on appearance of tumor recurrence (*p* = 0.289). Median recurrence-free survival (RFS) of the resection group was 9.7 months with a consecutive 1-, 3-, and 5-year RFS of 38, 16, and 12%, respectively.

#### Recurrence-free survival

**Fig. 1:**
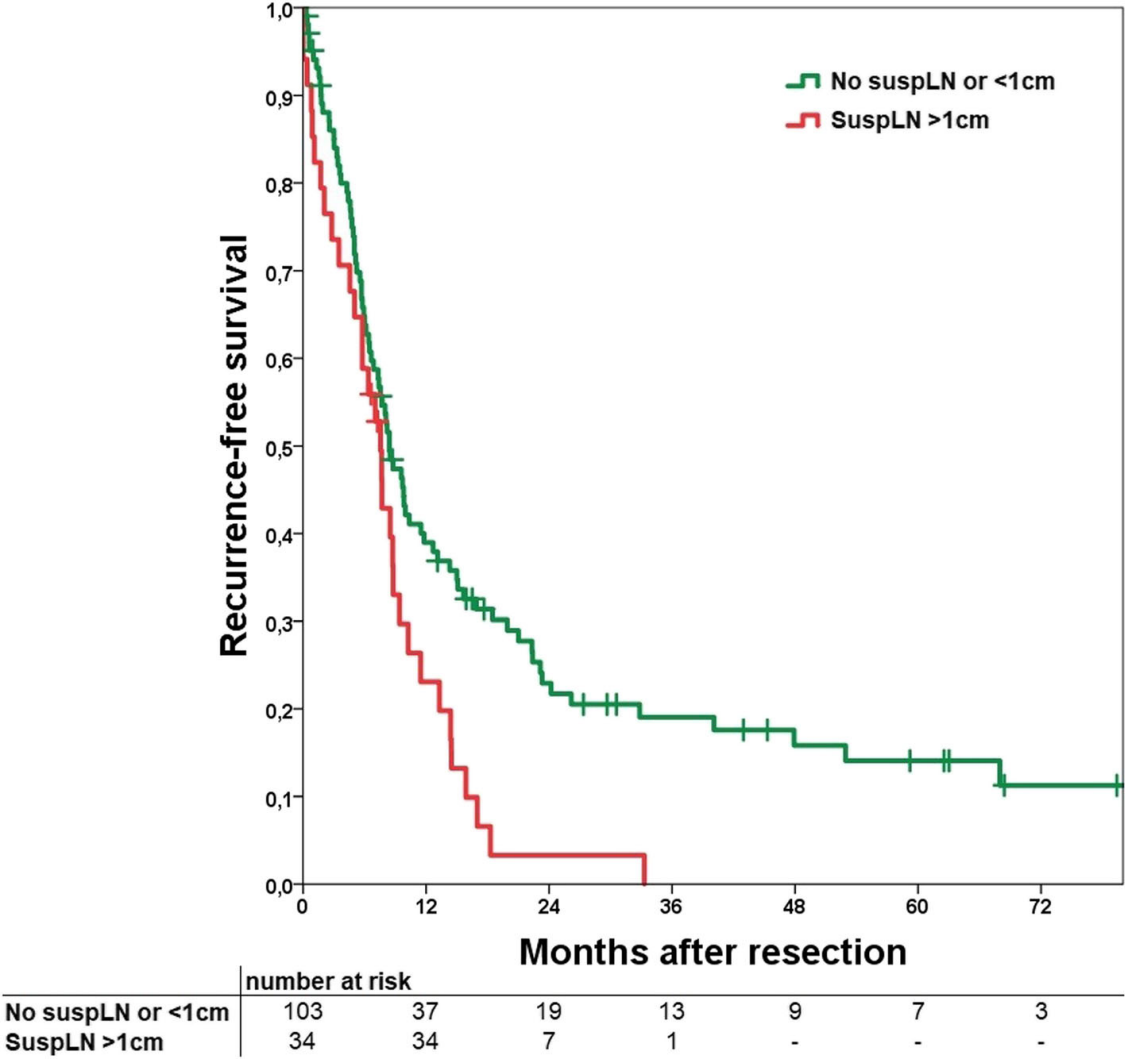
Kaplan-Meier curve of recurrence-free survival comparing a group of no suspicious lymph nodes or a diameter <  1 cm (No suspLN or <  1 cm) and suspicious lymph nodes with a diameter >  1 cm. *p* = 0.008

If recurrence-free survival (RFS) is calculated for positive versus negative suspLN no significant difference can be detected (*p* = 0.158). If a cut-off of 1 cm is applied, the group without suspLN had significantly better RFS compared to the > 1 cm group (*p* = 0.008; Fig. 1) with medians of 10.2 versus 8.4 months and a consecutive 1-, 3- and 5-year RFS of 41, 21, and 15% versus 29, 0, and 0%, respectively. Further subgroup analyses of the different lymph node stations showed no significant difference except the hepatoduodenal ligament (*p* = 0.036).

#### Overall survival

**Fig. 2:**
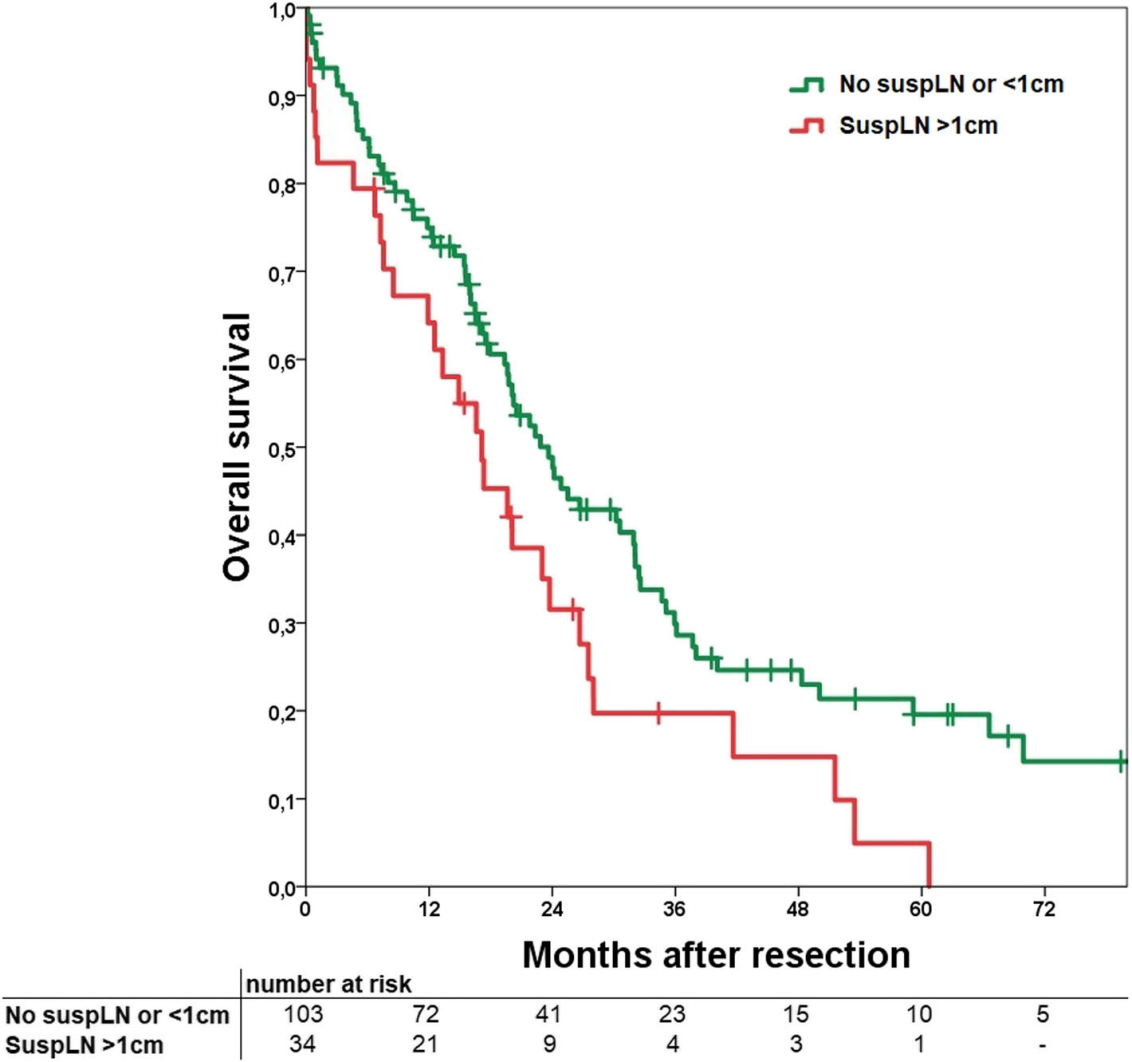
Kaplan-Meier curve of overall survival comparing a group of no suspicious lymph nodes or a diameter <  1 cm (No suspLN or <  1 cm) and suspicious lymph nodes with a diameter >  1 cm. *p* = 0.026

In analyses of OS, no difference can be shown for positive versus negative suspLN (*p* = 0.252) and different size categories (*p* = 0.067). Using a cut-off of 1 cm, median OS was 23.1 months versus 16.2 months with consecutive 1-, 3- and 5-year OS of 75, 30, and 18% versus 59, 18, and 6%, respectively (*p* = 0.026; Fig. 2). In further subgroup analyses for OS of the different lymph node stations with a 1 cm cut-off the hepatoduodenal ligament (*p* = 0.020) and retropancreatic (*p* < 0.001) were significantly different.

## Discussion

In this study we were able to show that suspLN in specific lymph node stations make irresectability more likely showing a significant relationship. Especially all tumors with suspLN superior to the diaphragm were irresectable, even if the number was small (*n* = 3). SuspLN within the hepatoduodenal ligament, retropancreatic or the lesser curvature were significantly associated with histopathological positive lymph node status. Sensitivity of suspLN was 71.1% and specificity 90.8%. An association of suspLN and tumor recurrence of any kind could not be shown. Using a cut-off of 1 cm short-axis diameter, suspLN had a significant influence on RFS and OS.

ICC is the second most common primary malignant tumor of the liver and often diagnosed in an advanced tumor stage due to a late onset of symptoms. The number of negative laparotomies varies depending on the aggressiveness of each surgical team, but is still high with 25–50% [7, 8, 12, 25]. To spare patients of this dilemma, a better preoperative evaluation with a precise assessment of resectability is essential. R0-resection remains the only chance of cure, but extrahepatic spread and often lymphatic spread is an obvious reason to resign from surgery. The preoperative evaluation of lymph nodes through imaging is always a vivid discussion in interdisciplinary tumor conferences regarding possibility and reasonableness of resection. We have an aggressive attitude towards resections to offer most patients the chance of complete tumor removal. If major or extended resections were performed in cases with suspected distant lymph node metastases was based on multiple considerations. In elderly or patients with a moderate state of health with a high risk of morbidity/mortality we would perform frozen section of clinically suspicious distant lymph nodes and resign from resection if positive. In younger patients or ones in good general state of health we would perform resection with extended lymphadenectomy because some of these patients will achieve long-term survival. Different studies within the last decade demonstrated that lymph node metastases are a predictor of poor OS and RFS for ICC keeping in mind that results and significance vary [2–8]. We aimed to demonstrate the relevance of preoperatively assessed lymph nodes via CT or MRI in five different typical lymph node stations regarding resectability, recurrence, OS, and RFS in patients with ICC.

Literature on preoperative evaluation of lymph nodes for ICC is scarce. Analyses of the influence of suspicious lymph nodes on resectability are not available. The ratio of suspLN in the exploration group divided by the total number of suspLN per lymph node station was calculated. A tendency becomes apparent that the likelihood of unresectability increases with suspLN in more distant lymph node stations. The fact that all tumors with suspLN superior to the diaphragm were unresectable because of intraabdominal tumor extension might be falsified due to the small number of patients in this subgroup, but nonetheless it is comprehensible. These patients might be candidates to resign from surgery or at least start with a laparoscopy, which has proven to be effective in ICC to minimize operative trauma and reduce the time until palliative treatment can be initiated [26, 27].

Suspicious lymph nodes in preoperative imaging are not automatically lymph node metastases. First of all it is important to say that lymphadenectomy is discussed intensively for ICC leading to a recommendation of harvesting six lymph nodes [1]. Analyses most often come to the conclusion that the number of metastatic lymph nodes is more important than the number of totally harvested lymph nodes [9–11]. The importance and influence especially of extended lymphadenectomy is not much considered for ICC yet. But for example, in perihilar cholangiocarcinoma it leads to worse long-term outcome and higher morbidity compared to regular lymphadenectomy and does not seem reasonable [28]. It is important to bring to mind that a suspicious lymph node in preoperative imaging is not always harvested routinely. Therefore, an inaccuracy remains in comparison of preoperative imaging and final histopathological results. The positive predictive value (PPV) between imaging and histology is not persuasive. Noji and colleagues focussed on this topic (via CT) and found a PPV of 56% for metastatic lymph nodes if the diameter of a round lymph node exceeded 16 mm [17]. This applies for MRI as well [29]. Other groups made comparable experiences that suspected lymph nodes are not associated with histopathologically proven metastases [30] or concluded that the preoperative evaluation remains difficult [18]. Park and colleagues showed a higher sensitivity (80%) and specificity (92.3%) in predicting lymph node metastases with preoperative PET-CT [31], while Yoh and colleagues were able to show that patients with a low CA19–9 value (< 37 IU/ml), peripheral ICC and no suspicious lymph nodes in preoperative imaging were likely to have no lymph node metastases (false negative rate 2.3%) [20]. We harvested a median of five lymph nodes, which gets close to the recommended amount of six in the AJCC/UICC/TNM classification [1].

Presence of cirrhosis influences size and shape of lymph nodes. In our study 12 patients were diagnosed with cirrhosis. In three of these patients we resigned from resection because of incidental finding of a before unknown cirrhosis, nine patients underwent minor resections. Only two of these patients had suspLN in the preoperative imaging, one in the resection and one in the exploration group. Therefore, we think it is safe to say that presence of cirrhosis did not affect our analysis.

Other clinicopathological parameters were also tested with suspicious lymph nodes and T status showed a significant correlation to N status while UICC stage significantly correlated with suspLN. That UICC stage and N status were significant as well can be easily explained because N1 automatically leads to an UICC stage of IIIb. Parameters like tumor size, multifocality or number of nodules showed no significant correlation which might have different reasons. On the one hand it is possible that the sample size is too small for example for number of nodules which nearly reaches significance with *p* = 0.087. On the other hand, tumor biology might not be defined through tumor size for example. A solitary ICC with a diameter of 8 cm might be less aggressive than three nodules with largest diameters of 3 cm. But this is prospect of ongoing investigations, not part of this study to answer and might be better understood in the future with other procedures like next generation sequencing (NGS).

SuspLN did not influence tumor recurrence of any kind in our cohort. This is comprehensible with the pattern of recurrence of ICC, which appears most often intrahepatic with only small amounts of extrahepatic or combined intra- and extrahepatic recurrences [13, 32, 33].

Survival analyses regarding suspicious lymph nodes show diverse results. Adachi and colleagues were able to show a significant benefit for the group without suspicious lymph nodes in preoperative imaging (patients with histologically proven lymph node metastases included) [18], while Marubashi and colleagues [30] did neither find an influence on OS nor RFS in patients with or without suspicious lymph nodes. Other articles did not analyse influence on survival. Our data suits perfectly in this diverse picture. For RFS, an influence could only be shown if a cut-off of 1 cm was used (*p* = 0.008); the same applies for OS (*p* = 0.026). This might show that lymph nodes below a short-axis diameter of 1 cm are not conclusive at all, even if they are round or with inhomogeneous vascularity. It is also according to our finding that with increasing size of suspicious lymph nodes the likelihood of metastases raises. All lymph nodes > 1.5 cm had metastatic disease (N+) except in one patient in interaortocaval position.

Single-center data with a consistent stem of surgeons led to a high homogeneity in decision making and comparable attitude. The number of 187 analysed patients with 137 resections is decent from our point of view. However, this study has several limitations. First of all, we want to address the retrospective design. Because of this it was not possible to react on findings of preoperative imaging and maybe extend lymphadenectomy to get a better picture of suspicion and histopathology. A multi-center study offers more patients and therefore more validity, even if it brings bias and negative features as well. Especially subgroup analyses are lacking of power due to the small number of patients. Another limitation is the correct diagnosis of lymph node metastasis. It is well known, that the correct classification of lymph node metastasis in cholangiocarcinoma and also most other types of cancer can be challenging as already stated above. Therefore, in this project the dedicated imaging analysis was carried out by an experienced investigator applying prespecified objective criteria as stated in the methods section. Nevertheless, misclassifications cannot be avoided. A technique which would have possibly improved detection rates to a certain amount is PET-CT; however this is not routinely performed in this tumor entity and is currently not recommended outside of clinical trials [19].

## Conclusions

In conclusion we were able to demonstrate that suspicious lymph nodes in preoperative imaging have influence on different factors. Resectability is more likely if the patient does not present with suspicious lymph nodes, but even if nodes > 1.5 cm are detected, resection and complete tumor clearance is still achievable. With a cut-off of suspicious lymph nodes of 1 cm a significant influence on RFS can be seen. The same applies for OS which is affected at a cut-off of 1 cm as well. It is important to classify these results in the correct way. Although suspicious lymph nodes in the preoperative imaging may be associated with a worse prognosis, in a considerable number of cases they are false positive. Explorative laparoscopy should be considered, especially if suspicious lymph nodes in distant localisations are detected in preoperative imaging to reduce operative trauma in case of irresectability. Surgery is the only chance of cure and therefore suspicious lymph nodes should be no reason to resign from surgery in most cases, because complete resection can still be achieved.

## Data Availability

The datasets used and analyzed during the current study are available from the corresponding author on reasonable request.

## Abbreviations

CT: Computed tomography
ICC: Intrahepatic cholangiocarcinoma
IQR: Interquartile range
MRI: Magnetic resonance imaging
OS: Overall survival
PET-CT: Positron emission tomography-computed tomography
RFS: Recurrence-free survival
suspLN: Suspicious lymph nodes
